# Electrocardiographic Findings in Brazilian Adults without Heart Disease: ELSA-Brasil

**DOI:** 10.5935/abc.20170146

**Published:** 2017-11

**Authors:** Marcelo Martins Pinto Filho, Luisa C. C. Brant, José Luiz Padilha-da-Silva, Murilo Foppa, Paulo A. Lotufo, José Geraldo Mill, Paulo R. Vasconcelo-Silva, Maria da Conceição C. Almeida, Sandhi Maria Barreto, Antônio Luiz Pinho Ribeiro

**Affiliations:** 1 Universidade Federal de Minas Gerais (UFMG), Belo Horizonte, MG - Brazil; 2 Universidade Federal do Rio Grande do Sul (UFRS), Porto Alegre, RS - Brazil; 3 Universidade de São Paulo (USP), São Paulo, SP - Brazil; 4 Universidade Federal do Espírito Santo (UFES), Vitória, ES - Brazil; 5 Universidade Federal do Estado do Rio de Janeiro (UNIRIO), Rio de Janeiro, RJ - Brazil; 6 Centro de Pesquisas Gonçalo Moniz da Fundação Oswaldo Cruz (FIOCRUZ), Salvador, BA - Brazil

**Keywords:** Electrocardiography/diagnosis, Adult, Epidemiology Measurements, Healthy People Programs, Cohort Studies

## Abstract

**Background:**

The electrocardiogram (ECG) is widely used in population-based studies. However, there are few studies on electrocardiographic findings in Latin America and in Brazil. The Brazilian Longitudinal Study of Adult Health (ELSA-Brasil) comprised 15,105 participants (35-74 years) from six Brazilian capitals.

**Objectives:**

To describe electrocardiographic findings in Brazilian adults without heart disease, stratified by sex, age and race/skin color.

**Methods:**

Cross-sectional study with baseline data of 11,094 adults (44.5% men) without heart disease from ELSA-Brasil. The ECGs were recorded with the Burdick Atria 6100 machine and stored at the Pyramis System. ECG analysis was automatically performed using the Glasgow University software. A descriptive analysis of heart rate (HR), P, QRS and T waves’ duration, PR and QT intervals, and P, R and T axes was performed. After stratification by sex, race/color and age, the groups were compared by the Wilcoxon and Kruskal-Wallis test at a significance level of 5%. Linear regression models were used to evaluate the behavior of electrocardiographic parameters over age. Major electrocardiographic abnormalities defined by the Minnesota code were manually revised.

**Results:**

Medians values of the electrocardiographic parameters were different between men and women: HR 63 vs. 66 bpm, PR 164 vs.158 ms, QT corrected 410 vs. 421 ms, QRS duration 92 vs. 86 ms, P-wave duration 112 vs. 108 ms, P-wave axis 54 vs. 57 degrees, R-wave axis 35 vs. 39 degrees, T-wave axis 39 vs. 45 degrees (p < 0.001 for all). The 2^nd^ and the 98^th^ percentiles of each variable were also obtained, and graphs were constructed to illustrate the behavior of the electrocardiographic findings over age of participants stratified by sex and race/skin color.

**Conclusions:**

The values for the electrocardiographic measurements herein described can be used as reference for Brazilian adults free of heart disease, stratified by sex. Our results suggest that self-reported race/skin color have no significant influence on electrocardiographic parameters.

## Introduction

Electrocardiography (ECG) is a low-cost, widely available test used in cardiovascular assessment.^1^ For decades, ECG has been used in large epidemiological studies, in which many of its diagnostic and prognostic utility was defined and confirmed.^2-6^ Electrocardiographic findings and their relationship with heart disease (HD) have long been the object of study in white and African-American populations. However, studies in Latin-America, especially in Brazilian population are still scarce. Besides, there are few data available in the medical literature about normal ECG values including ECG measurements, intervals, axes and wave duration for the Brazilian population,^7^ particularly for those whose clinical data are available.

The Brazilian Longitudinal Study of Adult Health (ELSA-Brasil)^8^ is a multicentric, cohort study aimed to prospectively evaluate participants’ health and detect determining factors for HD and diabetes. A comprehensive clinical data database of Brazilian adults was constructed from baseline examinations (2008-2010), and these data were correlated with their electrocardiographic parameters.^9^ Participants considered free of HD were selected for the present study.

The aim of the present study was to describe the duration of intervals and deflections in participants without HD selected from the ELSA-Brasil study. We aimed to establish normal values for electrocardiographic measurements by sex, age range and race/self-reported skin color in this population.

## Methods

### Participants

This study is a descriptive, cross-sectional analysis of data from ELSA-Brasil study, which aims at detecting HD and diabetes determinants in Brazilian adults. ELSA-Brasil study has been conducted in six capitals in Brazil - Belo Horizonte, Porto Alegre, Rio de Janeiro, Salvador, São Paulo e Vitoria - including 15,105 participants, using a methodology described elsewhere.^8-10^ ECGs from all participants were obtained during baseline examinations.

**Figure 1:**
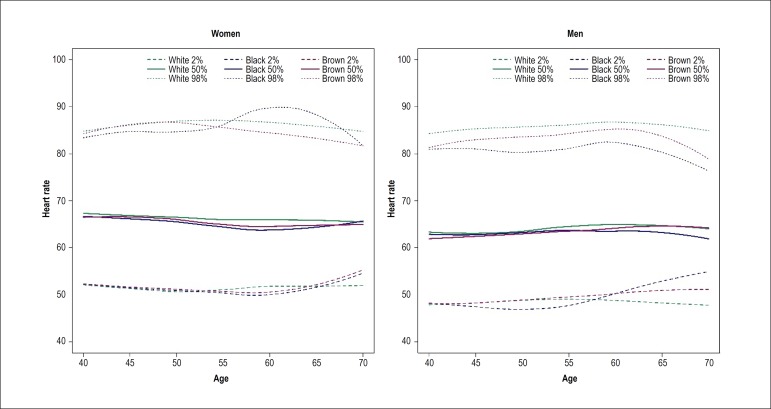
Heart rate by age in men and women stratified by self-reported race/skin color. The curves had a negative slope in women and a positive slope in men, with significant difference in pardo (brown race/skin color) men (p = 0.026), who showed lower variation in heart rate with age.

Participants with HD, those without race/skin color data (not declared) or of low-prevalent race (mainly of Asian or Indigenous origin), and participants with missing ECG data were excluded. A total of 11,985 participants were included in the study (Figure 1). A prevalent HD was defined as a self-reported history of severe coronary disease (history of acute myocardial infarction or myocardial revascularization), stroke, heart failure or major electrocardiographic changes, according to the Minnesota code (MC).

With respect to patients with systemic arterial hypertension (SAH), all analyses were performed twice - first including data from hypertensive patients’ and then excluding these data, in order to evaluate the impact of this comorbidity.

ELSA-Brasil was approved by the Ethics Committee from Universidade Federal de Minas Gerais, number ETIC 186/06, and performed according to the Helsinki declaration. All participants signed the informed consent form.

### ELSA-Brasil study protocol

From 2008 to 2010, participants were assessed using a standardized questionnaire, which included questions on cardiovascular system, and underwent anthropometric and physiological assessment including ECG. Risk factors for HD were defined according to national and international guidelines.^11,12^ SAH was defined by systolic blood pressure (SBP) ≥ 140 mmHg, diastolic blood pressure (DBP) ≥ 90 mmHg or use of antihypertensive drugs. Diabetes mellitus (DM) was defined by fasting glucose ≥126 mg/dL, postprandial glucose ≥ 200 mg/dL, or glycohemoglobin ≥ 6.5%, in addition to “being treated for DM” or having received the diagnosis of DM. Obesity was defined by a body mass index ≥ 30 Kg/m^2^, smoking was defined by “current smoking”, i.e., former smokers were not considered for this risk factor.

Race/skin color was assessed by self-report of participants, who answered a multiple-choice question according to the 2008 census in Brazil.^13^

### ECG testing

ECG was performed in each center following a previously established protocol,^9^ using a Burdick Atria 6100 device calibrated at 10 mm/mV and speed of 25 mm/second. Results were electronically transferred to a reading center located in Belo Horizonte and stored in an electronic database for posterior automated reading by the Glasgow ECG analysis program^14^ and codification by the MC.^15-17^ Acquisition and analysis of the ECGs are described in a previous publication^8^ and included established procedures of quality assurance procedures.

Measurements of PR and QT intervals, P-wave and QRS duration, and P, R and T axes were automatically performed. QT interval was corrected using the Hodges equation. ECGs were classified as presenting major, minor or no abnormalities according to the MC. For ‘major’ changes we considered: major Q (previous myocardial infarction MC 1-1, 1-2), minor Q plus major changes in ST-T segment (CM 1-3 and CM 4-1 or 4-2 or 5-1 or 5-2), major isolated ST-T abnormalities (CM 4-1 or 4-2 or 5-1 or 5-2), left ventricular hypertrophy associated with changes in ST-T segment (CM 3-1 and CM 4-1 or 4-2 or 5-1 or 5-2), intraventricular conduction defect (left bundle branch block, right bundle branch block, nonspecific intraventricular conduction delay, right bundle branch block associated with blockage of the anterior-superior division of the left bundle branch CM 7-1 or 7-2 or 7-4), Brugada ECG pattern (CM7-9), major QT prolongation (QT ≥ 116%), atrial fibrillation or atrial flutter (CM 8-3), supraventricular tachycardia (CM 8-4-2), atrioventricular conduction defect. (second- and third-degree block, pre-excitement and artificial pacemaker (CM 6-1 or 6-2 or 6-4 or 6-8), asystole and ventricular fibrillation (CM 8-2). Major ECG abnormalities were manually revised by experienced cardiologists for coding quality control and results were published elsewhere.^18^

### Statistical analysis

Categorical variables were described as frequencies (percentages). The Shapiro-Wilk test was used to test normality of data distribution; continuous variables with normal distribution were presented as mean and standard deviation, and those without normal distribution were expressed as median and percentiles. For representation of normality values, we used the 2^nd^ and 98^th^ percentiles in place of interquartile range. The percentiles relevant for ECG measures were calculated by age and their progress is shown by smoothed curves, using the loess method.

The Mann-Whitney and the Kruskal-Wallis tests were used for between-group comparisons (sex and race/skin color). The Bonferroni correction was used for multiple comparisons.

The inclusion of hypertensive participants was performed after a sensitivity analysis to evaluate the impact of this variable on the results.

In order to evaluate whether the slopes of the lines in the graph ECG measurements by age were similar between participants, we included interaction terms in liner regression models. ‘White’ race/skin color was used as reference due to the greatest number of individuals self-reported as so.

The level of significance was set at 5% unless stated otherwise. The analyses were performed using the SPSS version 20 and R version 3.3.0.

## Results

### Clinical characteristics of participants

Clinical characteristics of participants, stratified by sex and race/skin color, are described in Table 1. In general, there was a higher prevalence of SAH, smoking and DM in men than in women, whereas dyslipidemia and obesity were more prevalent among women. In the stratified analysis by self-reported race/skin color, SAH, DM and obesity were more prevalent in “black” race/skin color in both sexes.

**Table 1:** Characteristics of participants with electrocardiogram recordings at baseline, without evidence of heart disease (based on clinical history or electrocardiography test) (n = 11,985)

	Men (n = 5,341)			Women (n = 6644)		
Characteristics*	White (n = 2928)	Brown (n = 1672)	Black (n = 741)	White (n = 3577)	Brown (n = 1872)	Black (n = 1195)
Age	52(9)	50(8)	51(8)	52(9)	51(8)	51(8)
Heart rate	65(10)	63(10)	63(9)	67(9)	67(9)	66(9)
Systolic arterial pressure (mmHg)	122(14)	130(17)	130(17)	114(15)	118(16)	122(17)
Diastolic arterial pressure (mmHg)	78(10)	81(11)	81(10)	72(10)	75(10)	77(10)
Body mass index (kg/m^2^)	27(4.2)	27(4.2)	27(4.3)	26(4.9)	27(4.9)	28(5.5)
Fasting glucose (mg/dl)	114(29)	114(32)	119(40)	105(21)	108(28)	110(29)
LDL-cholesterol (mg/dl)	132(34)	132(37)	134(40)	131(34)	133(34)	129(35)
HDL-cholesterol (mg/dl)	50(11)	50(12)	54(14)	62(15)	61(14)	62(15)
Total cholesterol (mg/dl)	213(42)	214(47)	217(45)	217(40)	218(41)	212(43)
Hypertension (%)	32.7	36.4	45.6	23.8	30.2	43.1
Diabetes (%)	18.7	21.8	26.5	11.6	15.3	22.7
Dyslipidemia (%)	46.6	44.1	43.5	49.5	52.9	47.6
Obesity (%)	19.4	18.7	22.0	20.0	24.4	33.1
Smoking (%)	12.7	15.7	15.5	12.3	11.4	13.5

(*) continuous variables expressed as mean and standard deviation and categorical variables as percentage.

### Measurement of electrocardiographic intervals and deflections

Significant differences between men and women were found in all electrocardiographic parameters. Heart rate (HR) and QT duration were higher among women, whereas longer P-wave duration, QRS complex and PR interval were found in men (Table 2).

**Table 2:** Duration of electrocardiogram intervals and waves in men and women

Measurements*	Men (n = 5341)	Women (n = 6644)	p values (†)
Heart rate (bpm)	63(47 - 86)	66(51 - 87)	< 0.001
P-wave duration (ms)	112(78 - 134)	108(74 - 130)	< 0.001
PR interval (ms)	164(118 - 216)	156(114 - 208)	< 0.001
QRS duration (ms)	92(74 - 114)	86(70 - 106)	< 0.001
QT corrected (Hodges)(ms)	410(379 - 451)	421(389 - 459)	< 0.001
P-wave axis (degrees)	54(-11 - 77)	57(-10 - 78)	< 0.001
R-wave axis (degrees)	36(-43 - 84)	44(-29 - 84)	< 0.001
T-wave axis (degrees)	39(-14 - 77)	46(-07 - 77)	< 0.001

(*) Median and 2nd and 98th percentiles;

(†) Mann-Whitney test.

Sensitivity analysis that compared patients with and without SAH revealed no clinically important difference between the groups. Since there were an expressive number of hypertensive patients in the study, we decided to include these individuals in the final analysis. When these patients were excluded from the analyses, the results were quite similar to those obtained from the total study population (supplemental table 1 in apendix).

### Effect of race/skin color on electrocardiographic parameters

In the comparison of ECG measurements between races/skin colors, there was a statistically significant difference for most of the outcomes, except for R-wave axis for men and QT and P-wave axis for women. These differences are described in detail in Table 3.

**Table 3:** Duration of electrocardiogram intervals and waves by sex and race

	**Men**				
Measurements *	**White (1) (n = 2928)**	**Brown (2) (n = 1672)**	**Black (3) (n = 741)**	**p values (†)**	**Differences**
Heart rate (bpm)	64(47 - 86)	63(48 - 87)	63(46 - 84)	0.002	1 ≠ (2 = 3)
P-wave duration (ms)	112(78 - 136)	114(78 - 136)	114(80 - 137)	< 0.001	3 ≠ (1 = 2)
PR interval (ms)	164(118 - 216)	164(118 - 219)	166(124 - 225)	0.022	3 ≠ 1
QRS duration (ms)	92(74 - 114)	92(74 - 112)	92(72 - 112)	0.012	1 = 2 = 3
QT corrected (Hodges) (ms)	411(381 - 453)	410(377 - 449)	409(374 - 453)	0.008	2 ≠ 1
P-wave axis (degrees)	54(-10 - 77)	54(-13 - 77)	56(-7 - 79)	< 0.001	3 ≠ (1 = 2)
R-wave axis (degrees)	36(-44 - 83)	35(-42 - 85)	34(-41 - 82)	0.912	
T-wave axis (degrees)	40(-12 - 78)	37(-17 - 77)	34(-24 - 79)	< 0.001	1 ≠ (2 = 3)
	**Women**				
Measurements *	**White (1) (n = 3577)**	**Brown (2) (n = 1872)**	**Black (3) (n = 1195)**	**p values †**	**Differences**
Heart rate (bpm)	66(51 - 87)	66(50 - 87)	65(49 - 88)	0,019	3 ≠ 1
P-wave duration (ms)	108(72 - 130)	108(74 - 132)	108(74 - 133)	< 0.001	3 ≠ (2 = 1)
PR interval (ms)	156(114 - 208)	158(114 - 210)	160(118 - 216)	< 0.001	3 ≠ (2 = 1)
QRS duration (ms)	86(70 - 106)	86(70 - 106)	84(70 - 104)	< 0.001	1 ≠ (3 = 2)
QT corrected (Hodges) (ms)	421(389 - 459)	421(390 - 460)	420(385 - 462)	0.051	
P-wave axis (degrees)	57(-11 - 78)	56(-8 - 77)	56(-5 - 77)	0.050	
R-wave axis (degrees)	45(-33 - 84)	41(-25 - 83)	38(-24 - 80)	< 0.001	1 ≠ 2 ≠ 3
T-wave axis (degrees)	47(-4 - 77)	45(-16 - 78)	41(-20 - 76)	< 0.001	1 ≠ 2 ≠ 3

(*) Median and 2^nd^ and 98^th^ percentiles

(†) p-values calculated by the Mann-Whitney test; when statistically significant (p < 0.05), p-values between race groups were readjusted using the Bonferroni correction method, and considered significant when p < 0.0166.

**Figure 2:**
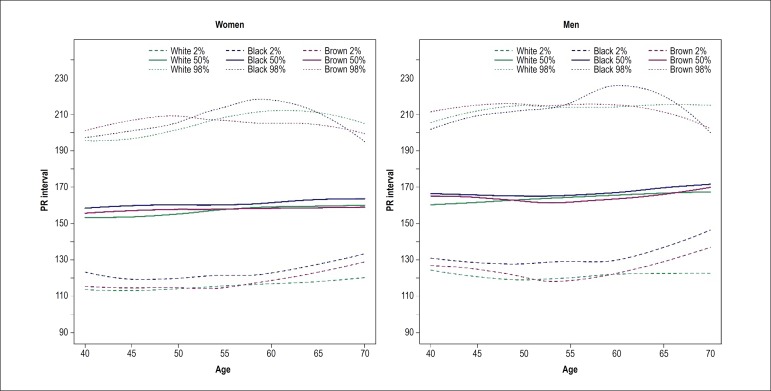
Duration of PR interval by age in men and women stratified by self-reported race/skin color. The curves have similar, positive slope, except for pardo (brown race/skin color) men, in which the slope is near zero, tending to negative (p = 0.032).

**Figure 3:**
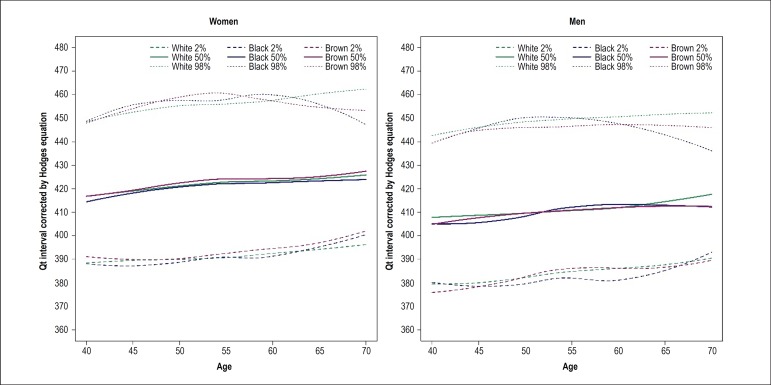
Duration of QT interval corrected by Hodges equation by age in men and women stratified by self-reported race/skin color. The slopes of the curves were positive, with no difference between them.

**Figure 4:**
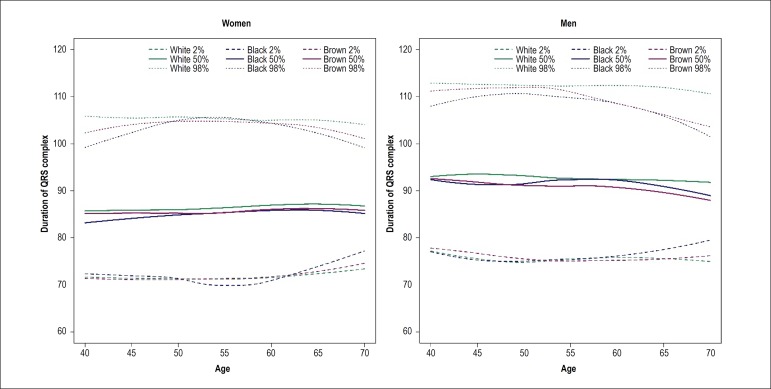
Duration of QRS complex by age in men and women stratified by self-reported race/skin color. The slopes were positive in women and negative in men, and significantly greater in pardo (brown race/skin color) men (p = 0.034).

**Figure 5:**
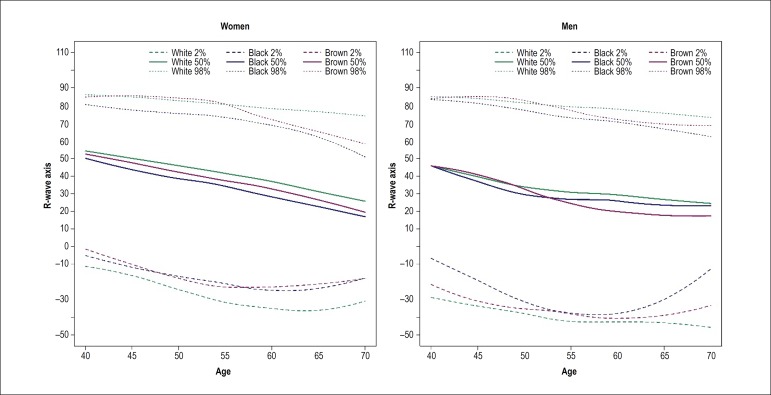
R-wave axis by age in men and women stratified by self-reported race/skin color. All curves had a negative slope; a significant difference was found only in pardo (brown race/skin color) men in which a significantly greater slope (p = 0.020) was observed.

In the graphs of ECG outcome by race/skin color stratified by sex, there was not a wide variation of HR over age in white individuals, whose median HR was slightly higher than that of other races/skin colors (Figure 1). PR interval also showed a slight increase with age, and black race/skin color median line was constantly greater over age than median lines of other races/skin colors in both sexes (Figure 2). QTc interval (QT corrected by the Hodges equation) increased with age and was more prolonged in women than in men in all ages (Figure 3). QRS duration was relatively constant with age, with higher median values in white race/skin color in both sexes (Figure 4). There was also a decrease in R-wave axis with age, with higher median values in white race/skin color at all age ranges, which was more evident in women (Figure 5). The behavior of P-wave duration, P-wave axis and T-wave axis can be analyzed in the Appendix (Figures 1, 2 and 3).

With respect to the slope values (variation of the measurements with age), there was no difference between races/skin colors, except for *pardo* (brown skin color) men, who showed lower HR variation and PR interval duration and greater variation of QRS complex and R-wave axis duration as compared with white race/color.

## Discussion

The present study enabled the description of electrocardiographic parameters of Brazilian adults of both sexes without HDs. This is the first publication to describe normal ECG parameters in the Brazilian population. Besides, although previous studies have been performed in many populations,^19-22^ most of them included smaller sample sizes, except for the study by Rijnbeek et al.,^22^ that included 13,354 participants aged between 16 and 90 years from four population studies in the Netherlands. These studies included apparently healthy subjects defined according to standardized questionnaires. Individuals using medications for HDs and those with risk factors for DM and SAH were excluded. In the present study, we chose not to exclude SAH patients without clear evidence of HD based on the assumption that excluding those participants with major electrocardiographic abnormalities (classified by the MC), we would exclude those hypertensive patients with significant electrocardiographic changes caused by SAH (e.g. left bundle branch block, ventricular hypertrophy with repolarization abnormalities). After excluding patients with SAH, sensitivity analysis revealed no clinically significant differences in the electrocardiographic parameters, which corroborated our decision not to exclude these patients from the analyses and gave power to our study.

In the analysis stratified by sex, we observed that QTc was consistently greater in men over different age ranges. The difference in median values was similar (approximately 10 ms) to those described in which different reference values by sex were used.^23^ Also, similar to our study, the authors did not report clinically significant differences in the other electrocardiographic parameters between the sexes.

Some differences can be pointed out between our results and others reported in a predominantly Caucasian sample^22^: our sample had lower median HR, and P-wave, PR interval and QRS duration. QT corrected by Hodges formula was not significantly different. Nevertheless, these measurements were higher than those reported in a study conducted in India.^21^

On the other hand, there were also similarities between the current study and previous reports. The increase trend of QT corrected and the R-wave axis deviation to the left with age were also reported in populations from different countries, including different races.^18,21^

Despite numerous studies investigating electrocardiographic parameters in different populations, there is still little evidence of the impact of race on these parameters.^19-22^ In our study, patients were stratified by race/skin color according to their own reports; only the most prevalent races were included in the analysis and participants who self-reported as “yellow” or “indigenous” were excluded. Although statistically significant differences were found in many parameters between different races/skin colors, the clinical significance of these findings remain questionable. Besides, these differences were of only milliseconds between wave intervals and durations, and there was considerable overlapping of the curves in the graphs.

Among the limitations of our study, we can mention the difficulty in analyzing race/skin color from participants’ own reports in such a mixed-race country as Brazil. In this context, distinction between white, *pardo* (brown) and black may be challenging. The decision to maintain hypertensive participants in the analysis of ECGs should be seen with caution, since the possibility that this comorbidity may have affected the results cannot be ruled out.

A strength of this study was the large sample size and the analysis of the relationship between race/skin color and ECG findings. These were obtained using devices of the same brand and model, and a uniform protocol. The clinical variables obtained in a standardized method and a strict quality control enabled a detailed characterization of each participant’s health status and clear identification of those free of HD.

From a practical standpoint, our findings tend to corroborate the use of traditional reference values, since they were similar to the results found in this Brazilian population. It is worth mentioning, however, that the interpretation of the PR interval should be viewed with caution, since variation of this parameter within the percentiles considered in the analyses was greater than 200 ms, which is currently considered the cutoff point for first-degree atrioventricular block.^24^

For future perspectives, we highlight the prospective nature of this study, which will make possible the assessment of the electrocardiographic changes in the participants and the effects of aging in this cohort in the outcomes measured. In the current scenario in which physicians try to provide patient-centered care based on patients’ needs, our findings will enable the interpretation of ECG in an individualized manner, with possible variations in age- and sex-specific reference values for the Brazilian population. As ECG reading programs and digital ECG devices improve, this scenario may be closer to reality.

## Conclusion

This is the first study conducted in Latin America, specifically in Brazil, on the influence of race/skin color on the electrocardiographic parameters. The ECG values here described can be used as reference values for Brazilian adults of both sexes without HD. Our results suggest that self-reported race/skin color had no significant influence on the electrocardiographic parameters.
